# Base editing in crops: current advances, limitations and future implications

**DOI:** 10.1111/pbi.13225

**Published:** 2019-08-15

**Authors:** Rukmini Mishra, Raj Kumar Joshi, Kaijun Zhao

**Affiliations:** ^1^ National Key Facility for Crop Gene Resources and Genetic Improvement (NFCRI), Institute of Crop Science Chinese Academy of Agriculture Sciences (CAAS) Beijing China; ^2^ Department of Biotechnology Rama Devi Women's University Bhubaneswar Odisha India

**Keywords:** adenine, base editors, cytidine, CRISPR/Cas9, crop improvement, genome editing

## Abstract

Targeted mutagenesis via genome‐editing technologies holds great promise in developing improved crop varieties to meet future demands. Point mutations or single nucleotide polymorphisms often determine important agronomic traits of crops. Genome‐editing‐based single‐base changes could generate elite trait variants in crop plants which help in accelerating crop improvement. Among the genome‐editing technologies, base editing has emerged as a novel and efficient genome‐editing approach which enables direct and irreversible conversion of one target base into another in a programmable manner. A base editor is a fusion of catalytically inactive CRISPR–Cas9 domain (Cas9 variants) and cytosine or adenosine deaminase domain that introduces desired point mutations in the target region enabling precise editing of genomes. In the present review, we have summarized the development of different base‐editing platforms. Then, we have focussed on the current advances and the potential applications of this precise technology in crop improvement. The review also sheds light on the limitations associated with this technology. Finally, the future perspectives of this emerging technology towards crop improvement have been highlighted.

## Introduction

**Figure 1:**
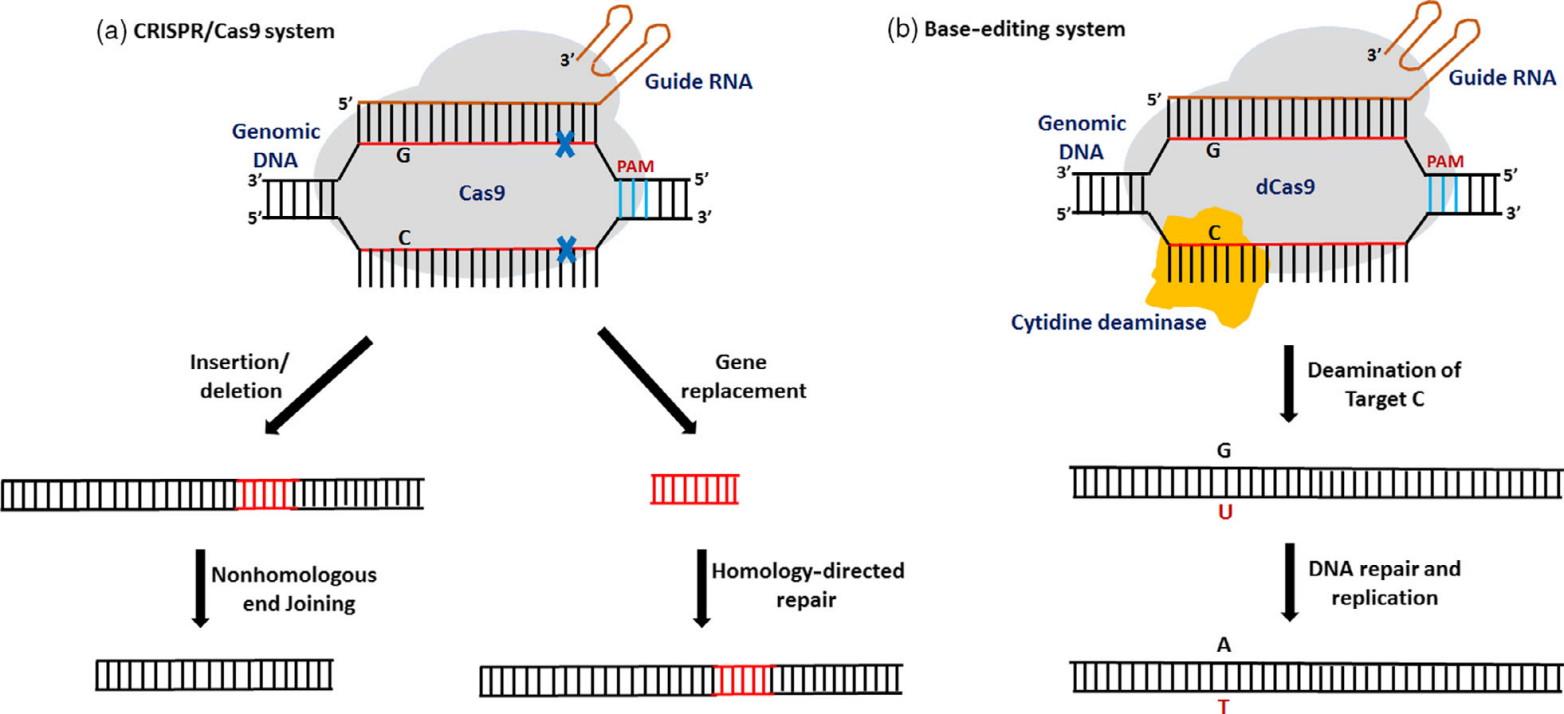
Comparative representation of the mechanism of CRISPR/Cas9 and a base‐editing system. (a) In a CRISPR/Cas9 system, the Cas9‐sgRNA complex moves along the DNA strand and makes a double‐stranded break (DSB) where the Cas9 encounters the appropriate protospacer adjacent motif (PAM) and the sgRNA matches the target DNA sequence. These DSBs are subsequently repaired either by nonhomologous end‐joining (NHEJ) or by homology‐directed repair pathway (HDR). (b) In a base‐editing system, a catalytically dead Cas9 endonuclease (dCas9) fused to a catalytic cytidine deaminase domain is guided by a sgRNA molecule to make single‐base substitutions without creating a double‐stranded break in the DNA.

Genome editing via the CRISPR/Cas9 system has flourished as an efficient technology and has revolutionized the field of agriculture and plant science with its simplicity, versatility and high precision. In a CRISPR/Cas9 genome‐editing system, the Cas9‐sgRNA complex moves along the DNA strand and makes a double‐stranded break (DSB) where the Cas9 encounters the appropriate protospacer adjacent motif (PAM) and the sgRNA matches the target DNA sequence (Jinek *et al.*, 2014). These DSBs are subsequently repaired by the naturally occurring DNA repair pathways: nonhomologous end‐joining (NHEJ) or homology‐directed repair pathway (HDR). NHEJ is an error‐prone repair pathway which results in random insertions and deletions whereas HDR is a high‐fidelity repair method which results in gene insertion or gene replacements (Voytas and Gao, 2014) (Figure 1). CRISPR/Cas9 system is a highly efficient and robust system used for genome editing and has been successfully used for genome editing in crops and model plants due to its adaptability and high precision (Liang *et al.*, 2017; Shao *et al.*, 2017; Shi *et al.*, 2017; Tomlinson *et al.*, 2019; Zsögön *et al.*, 2018) . However, unintended mutations in the off‐target regions and PAM specificity are still the major problems associated with this technology.

Many important agronomic traits are determined by point mutations or a few base changes in a gene (Doebley *et al.*, 2006; Li *et al.*, 2017; Ma *et al.*, 2015). CRISPR/Cas9‐mediated gene replacements via homology‐directed repair (HDR) has been reported as a feasible approach to correct the point mutations in the target gene and has the potential for accelerating crop improvement (Li *et al.*, 2018; Sun *et al.*, 2016; Wang *et al.*, 2017). However, infrequent occurrence of HDR and low efficiency of template DNA delivery have always been a challenging task in achieving success in plants (Ran *et al.*, 2017). Moreover, CRISPR/Cas9 system is suitable for gene knockout or knock‐in, but cannot convert one base into another. These limitations have highlighted the need for alternative approaches which can result in stable and precise genome editing in crops.

“Base editing” has emerged as a novel approach which enables precise nucleotide substitutions in a programmable manner, without disruption of a gene or requiring a donor template (Komor *et al.*, 2016). A base editor is a fusion of catalytically inactive CRISPR–Cas9 domain (Cas9 variants, dCas9 or Cas9 nickase) and a cytosine or adenosine deaminase domain which converts one base to another (Figure 1). Single‐base changes could generate elite trait variations in crop plants which help in accelerating crop improvement. The base‐editing system can revert a single‐base change or SNP without gene disruption, thereby minimizing the insertions and deletions. It is an efficient technology for engineering novel traits in agriculturally important crops and a key to food security (Eid *et al.*, 2018).

In the last 3 years, the cytosine and adenine base editors (ABEs) have emerged as efficient tools for precise genome modification (C to T or A to G) in eukaryotic genomes (Hua *et al.*, 2018; Liu *et al.*, 2018; Qin *et al.*, 2019; Zong *et al.*, 2017). Base‐editing approach has been efficiently optimized and demonstrated in several crops including rice, wheat, maize and tomato (Li *et al.*, 2018; Lu and Zhu, 2017; Tang *et al.*, 2019; Zong *et al.*, 2017). Numerous articles and a huge accumulation of case studies on base‐editing system and its application in crops have highlighted the need for an elaborative review which will be a valuable source of information for the scientific community. In the present review, we have summarized the development of different base‐editing platforms and their efficiencies in editing both DNA and RNA. The highlight of the review is the potential applications of base‐editing technology in crop improvement using specific case studies. The review will also discuss the limitations and the future implications of this novel emerging technology.

## CRISPR‐based base editors – overview

### DNA base editors

Base editors are chimeric proteins composed of a DNA targeting module and a catalytic domain which is capable of deaminating a cytosine or adenine base in the genome (Gaudelli *et al.*, 2017; Komor *et al.*, 2016). The DNA targeting module is either a catalytically dead Cas9 endonuclease (dCas9) or a Cas9 nickase guided by a sgRNA molecule. The dCas9 contains Asp10Ala and His840Ala mutations that inactivate its nuclease activity but retain the DNA binding ability. The binding of dCas9‐sgRNA to the target DNA creates an ‘R‐loop’ where a stretch of DNA gets unpaired. This small single‐stranded domain of approximately 5–8 nucleotides acts as an editing or catalytic window for dCas9‐tethered deaminase to modify the cytosines. The base editors are capable of making single‐base changes or substitutions without creating a DSB in the DNA, thereby limiting the frequency of indels. There are two types of DNA base editors: cytosine base editors (CBEs) and ABEs. The characteristics, catalytic window and functions of CBEs and ABEs have been listed in Table 1.

**Table 1:** List of base editors, characteristics, catalytic window and functions

Base editors	Characteristics	Type of base substitutions	Catalytic window	References
DNA base editors				
BE1	(APOBEC1–XTEN–dCas9): Composed of a cytidine deaminase enzyme APOBEC1 (from rats) linked to a catalytically dead Cas9 (dcas9) by a 16 amino acid XTEN linker	C to T	−17 to −13	Komor *et al*. (2016)
BE2	(APOBEC–XTEN–dCas9–UGI): UGI is fused to the C terminus of BE1.	C to T	−17 to −13	Komor *et al*. (2016)
BE3	(APOBEC–XTEN–Cas9n–GI): rAPOBEC1 fused to the N terminus of nickase cas9 D10A through a 16‐amino acid XTEN linker and a UGI fused to the C terminus by a 4‐amino acid linker	C to T	−16 to −12	Komor *et al*. (2016)
YEE‐BE3	(W90Y+R126E+R132E): triple mutant	C to T	−15 to −13	Kim *et al*. (2017)
BE4	Composed of rAPOBEC1 fused to Cas9D10A through a 32‐aa linker and two UGI molecules are linked to both C and N terminal of Cas9 nickase by a 9‐aa linker.	C to T	−17 to −13	Komor *et al.* (2017)
SaBE4‐GAM	Gam protein fused to *Staphylococcus aureus* Cas9‐derived BE4	C to T	−19 to −9	Komor *et al.* (2017)
Target‐AID	Composed of nickase Cas9D10A and a cytidine deaminase pmCDA1 (from sea lamprey)	C to T	−19 to −15	Nishida *et al.* (2016)
TAM	dCas9 is fused to human AID; co‐expressed with UGI	C to T	−16 to −12	Ma *et al*. (2016)
CRISPR‐X	dCas9 is used to target a hyperactive AID variant to induce localized, diverse point mutations. The sgRNA backbone contains two MS2 RNA hairpins that each recruit two MS2 proteins fused to AID	C to T	−50 to +50	Hess *et al.* (2016)
ABE	TadA is fused to a catalytically impaired CRISPR/Cas9 mutant	A to G	−17 to −14	Gaudelli *et al*. (2017)
RNA base editor				
ADAR	Catalytically inactive Cas13 (dCas13) is fused to a naturally occurring ADAR (adenosine deaminase acting on RNA)	A to 1	−50 to +50	Cox *et al*. (2017)

### Cytosine base editors

Cytosine base editors are the vectors that catalyse the conversion of cytosines to thymines. The cytidine deaminase enzyme removes an amino group from cytosine converting it to uracil, resulting in a U‐G mismatch which gets resolved via DNA repair pathways to form U‐A base pairs. Subsequently, a T gets incorporated in the newly synthesized strand forming T‐A base pairs. This results in C‐G to T‐A conversion in a programmable manner. The first‐generation base editor (BE1) was developed by David Liu and co‐workers of Harvard University, USA, in 2016. It was composed of a cytidine deaminase enzyme APOBEC1 (from rats) linked to a dCas9 by a 16 amino acid XTEN linker (Komor *et al.*, 2016). The XTEN is a peptide which links them and maintains a balance between the two proteins. The apolipoprotein B mRNA editing enzymes, catalytic polypeptide‐like (APOBEC) family are a group of naturally occurring cytidine deaminases in vertebrates which protect them from invading viruses (Chiu and Greene, 2006). These enzymes act on single‐stranded DNA/RNA as substrates.

**Figure 2:**
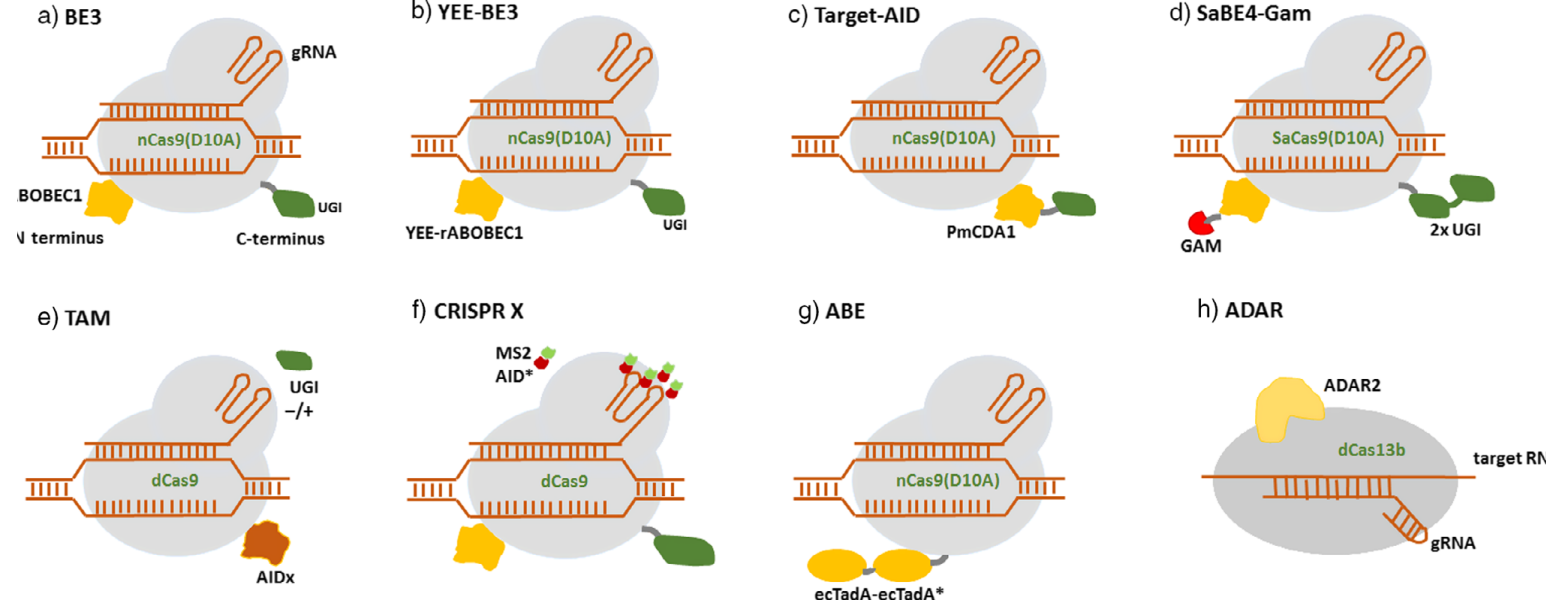
Structural representation of base‐editing platforms: (a) BE3 employs Cas9 nickase (nCas9D10A) along with a cytidine deaminase rAPOBEC1 (orange) and an uracil DNA glycosylase inhibitor (UGI) (Green). (b) YEE‐BE3 employs YEE‐rAPOBEC1. (c) Target‐AID employ PmCDA1 (d) SaBE4‐gam employs SaCas9D10A, 2 × UGI and has a Gam protein (red) fused to its terminus. (e) and (f) The TAM and CRISPR‐X systems used dCas9 to recruit variants of the deaminase AID (AIDx or MS2‐AID*D). (g) ABE is composed of ecTadA (WT)‐ecTadA* (7.10) heterodimer fused to Cas9n. (h) Catalytically inactive Cas13 (dCas13) is fused to a naturally occurring ADAR2 (adenosine deaminase acting on RNA).

The major limitation in BE1 was the frequent removal of uracil by uracil DNA glycosylase (UDG), resulting in low editing efficiency. Keeping in view the low editing efficiency and limitations of BE1, a series of improved base editors were developed further. The second‐generation base editor BE2 (APOBEC‐XTEN‐dcas9‐UGI) was developed by adding a uracil DNA glycosylase inhibitor (UGI) to the C terminus of the DNA targeting module (Komor *et al.*, 2016). The addition of UGI inhibits the activity of UDG that catalyses the removal of U from DNA in cells and initiates base excision repair (BER) pathway. UGI is an 83‐residue protein from *Bacillus subtilis* bacteriophage PBS1 which blocks UDG activity in human cells. This inhibition of BER increases the editing efficiency by threefold in human cells. Subsequently, BE3 base editor was developed, which was composed of rAPOBEC1 fused to the N terminus of nickase cas9 D10A through a 16‐amino acid XTEN linker and a UGI fused to the C terminus by a 4‐amino acid linker (Komor *et al.*, 2016；Figure 2a). The major improvement in BE3 was the replacement of dCas9 with Cas9 nickase (nCas9), which nicks the strand opposite to the deaminated cytidine. dCas9 is converted to nCas9 either by replacing amino acid aspartate (D) by alanine (A) at position 10 (D10A) or by replacing histidine (H) by alanine at position 840 (H840). The nick initiates a long‐patch BER, where the deaminated strand is used as a template to produce U‐A base pair, further converted to T‐A during DNA replication. Thus, the editing efficiency was further increased by sixfold in BE3 over BE2. The use of nCas9 also exhibited an increase in indel frequency of 1.1 % as compared to 0.1 % in BE2.

**Figure 3:**
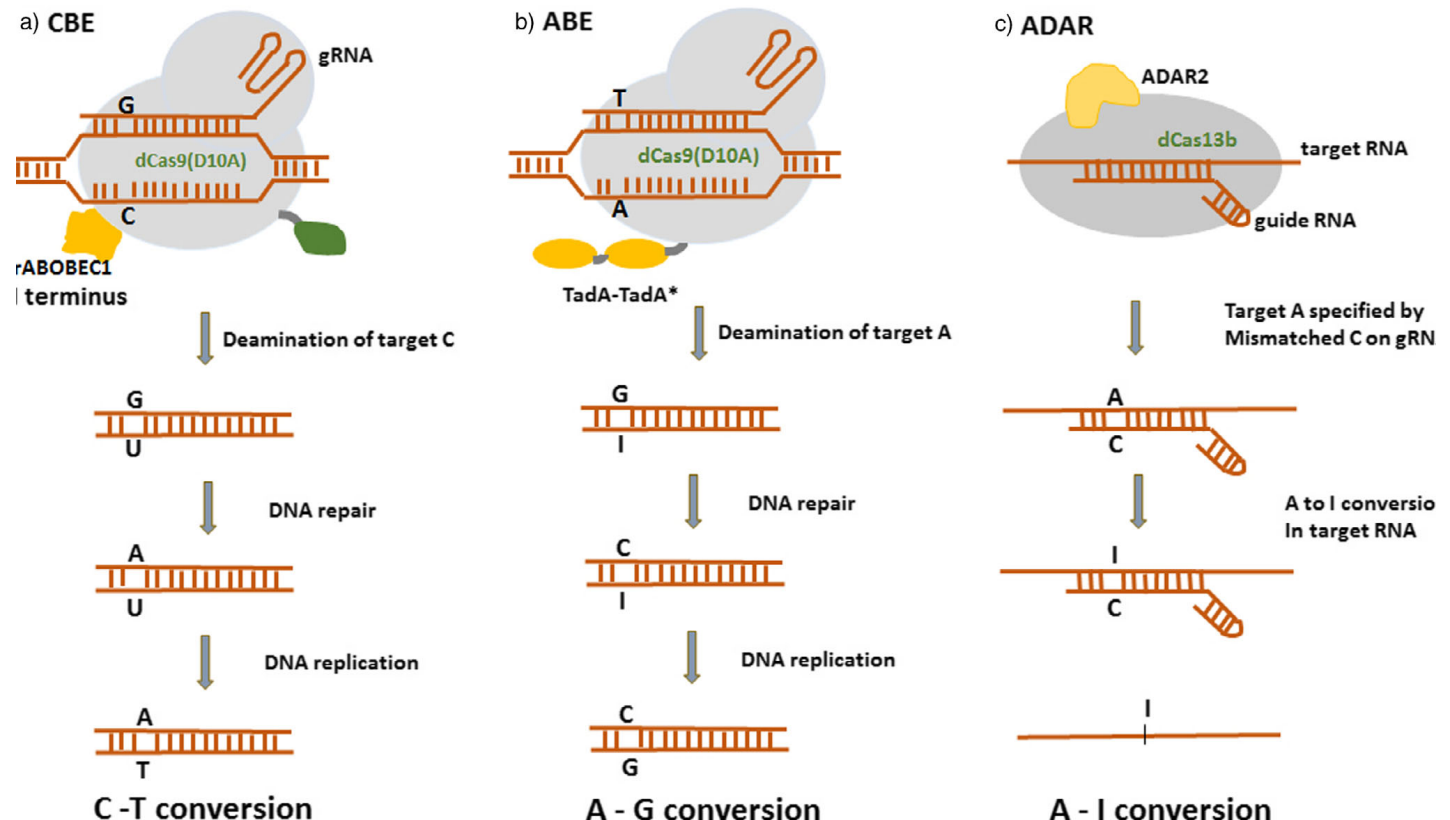
A comparison of three different approaches of base editing. (a) CBE – mediated base‐editing strategy results in C‐T conversions. (b) ABE – mediated base‐editing strategy results in A‐G conversions. (c) ADAR – mediated RNA base‐editing results in A‐1 conversion.

Cytosine base editors enable C‐G to T‐A conversion in a programmable manner (Figure 3a). However, the occurrence of more than one cytosines (Cs) within the catalytic window may result in off‐target activity and conversion of nontarget C to U. To overcome this limitation, several BE3 variants were generated with different Cas9 variants (using noncanonical PAM). The SpCas9 variants like VQR‐BE3, EQR‐BE3, VRER‐BE3 and SaKKH‐BE3 which target NGAN, NGAG, NGCG and NNNRRT PAMs, respectively, have increased the editing efficiency by 2.5‐folds (Kim *et al.*, 2017). Besides SpCas9 variants, SaCas9 (from *Staphylococcus aureus*), with NNGRRT PAM, has been used in several studies with enhanced efficiency. Several cytidine deaminase mutants like YEE‐BE2 and YEE‐BE3 with varying editing window widths were generated to enhance DNA specificity and reduce off‐target editing. The triple mutant W90Y+R126E+R132E (YEE‐BE3) exhibited maximal editing efficiencies within a narrow editing window width of approximately 2 nucleotides (Kim *et al.*, 2017) (Figure 2b).

Another base‐editing system, Target‐AID (activation‐induced cytidine deaminase), was developed which was composed of a nickase Cas9D10A and a cytidine deaminase pmCDA1 (from sea lamprey) (Nishida *et al*., 2016) (Figure 2c). AID causes deamination of cytidine and protects the vertebrate cells from foreign invaders by altering their genomes, facilitating somatic hypermutation and class switch recombination in vertebrates (Nishida *et al.*, 2016). It targets the immunoglobulin (Ig) locus and generates diverse mutations that are selected through antigen binding. AID deficiency causes hyper‐IgM syndrome which generates low‐affinity antibodies (Revy *et al.*, 2000; Xu *et al.*, 2012). Thus, the target‐AID system was used to perform targeted mutagenesis with improved efficiency in mouse and human cells. The use of nickase and UGI has enhanced the editing efficiency by twofold to threefold in BE3 and Target‐AID.

To further expand and increase the base‐editing efficiency, fourth‐generation base editors BE4 (*S. pyogenes* Cas9‐derived base editor) and SaBE4 (*S. aureus* Cas9‐derived BE4) were developed by linking rAPOBEC1 to Cas9D10A through a 32‐aa linker and fusing two UGI molecules to both C and N terminal of Cas9 nickase by a 9‐aa linker (Komor *et al.,* 2017). The use of UGI blocks the access of UNG to the uracil intermediate, inhibiting BER, thereby minimizing the formation of undesired by‐products. Additionally, a double‐stranded DNA end‐binding protein Gam derived from bacteriophage Mu was fused to N terminus of a Cas9 nickase which binds to the free ends of DSB and reduces indel formation during base‐editing process, thereby improving product purity. BE4‐Gam and SaBE4‐Gam (Figure 2d) have exhibited an average decrease in non‐T product formation and increased C to T editing efficiency as compared to BE4 and SaBE4. Thus, fourth‐generation base editors were efficiently used for programmable C to T conversion with reduced indel formation and increased product purity.

Besides being used to introduce point mutation in a precise and programmable manner, deaminases are also used to create a diverse library of point mutations localized to a targeted region of the genome. Targeted AID‐mediated mutagenesis (TAM) and CRISPR‐X are the two DNA base‐editing platforms which have been used to generate localized sequence diversity through base editing (Hess *et al.*, 2016; Ma *et al.*, 2016) (Figure 2e,f). In the TAM system, dCas9 is fused to human AID which enables efficient genetic diversification in mammalian cells (Ma *et al.*, 2016). When co‐expressed with UGI, the mutation frequencies of dCas9‐AIDx were increased by five times and restricted the mutations strictly to C to T or G to A substitutions. Cas9‐AIDx is used to create a diverse mutation spectrum beyond the C to T or G to A substitutions and its independence of the AID hotspot motifs. TAM is established as an efficient genetic diversification strategy in mammalian cells to facilitate protein evolution which was not feasible earlier. The dCas9‐AID was stably expressed in K562 cells, a chronic myelogenous leukaemia (CML) line that contains the BCR‐*ABL* oncogene and is sensitive to imatinib. The TAM system is used to target BCR‐ABL to identify known and novel mutations conferring imatinib resistance in chronic myeloid leukaemia cells. Imatinib (Gleevec) inhibits ABL and other tyrosine kinases by binding to their catalytic domains and has become a standard therapy against BCR‐*ABL* + CML. TAM is an ideal platform to understand protein function and enables the identification of drug targets and new mechanisms of drug resistance.

In CRISPR‐X, the dCas9 is used to target a hyperactive AID variant to induce localized, diverse point mutations (Hess *et al.*, 2016). In this system, the sgRNA backbone contains two MS2 RNA hairpins that each recruit two MS2 proteins fused to AID. The AID exhibited a larger window of catalytic activities between −50 to +50 from the PAM sequence and induced a twofold to sixfold increase in mutation frequency. The CRISPR‐X system has been successfully used to induce mutagenesis of the target of the chemotherapeutic bortezomib (PSMB5), identifying novel drug‐resistant mutants that may reveal new properties of PSMB5 and its interaction with bortezomib (Hess *et al.*, 2016).

The dCas9 has been successfully used as a DNA targeting module for gene editing purposes. However, the requirement of G/C‐rich PAM sequences is still a limitation. In order to expand the scope of base editing, Li and colleagues have generated the first Cpf1‐based cytidine deaminase base editor (Li *et al.*, 2018). The Cpf1 is a type V class 2 CRISPR endonuclease. It favours T‐rich, ‐TTTN‐, PAMs, generates cohesive end with 5‐bp (Zetsche *et al.*, 2015) and can process its sgRNA which enhances its use in multiplex genome targeting (Zetsche *et al.*, 2017). The base editor, dLbCpf1‐BE0, is composed of a rat APOBEC1 domain fused to a catalytically inactive *Lachnospiraceae* bacterium Cpf1 (dLbCpf1) and UGI. The editing window of this base editor ranges from positions 8 to 13 bp preceding the PAM and exhibits an editing efficiency of 20–22%. Besides dLbCpf1‐BE0, Li *et al*. (2018) have generated other fusions dCpf1‐BE‐YE, dCpf1‐eBE and dCpf1‐eBE‐YE based on the Cas9‐BEs generated by Komor *et al*. (2016). Thus, the use of Cpf1 based base editors could extend the scope of base editing by providing various choices of PAMs in the target gene.

### Adenine base editors

Base‐editing capabilities and study of genetic diseases were further expanded by the development of a new class of ABEs that could modify adenine bases (Gaudelli *et al.*, 2017) (Figure 2g). Unlike cytidine deaminases, adenine DNA deaminases do not occur in nature. In 2017, David Liu and group developed ABEs by using *Escherichia coli* TadA (*E. coli* TadA) through extensive protein engineering and directed evolution. *E. coli* TadA is a tRNA adenine deaminase that converts adenine to inosine in the single‐stranded anticodon loop of tRNA Arg (Figure 3b). It shares homology with the APOBEC enzyme. The first‐generation ABEs were developed by fusing a TadA with a catalytically impaired CRISPR/Cas9 mutant (Gaudelli *et al.*, 2017). Among the series of ABEs developed, ABE7.7, ABE7.8 and ABE7.9 are considered to be the most active ABEs with a broader sequence compatibility. The seventh‐generation ABEs (ABE7.10) were recommended for conversion of A.T to G.C in a wide range of targets with increased efficiency and product purity. ABEs introduce point mutations with higher efficiency and have greatly expanded the scope of base editing by enabling all four transitions (C to T, A to G, T to C and G to A) in a programmable manner.

### RNA base editors (ADAR)

Feng Zhang and his group were the first to develop RNA base editors by using a catalytically inactive Cas13 (dCas13) and a naturally occurring ADAR (adenosine deaminase acting on RNA) to direct adenosine to inosine conversion in mammalian cells (Cox *et al.*, 2017) (Figure 2h). Cas13 is a type VI CRISPR‐associated RNA‐guided RNase with RNA binding abilities. Among a set of Cas13 enzymes assayed for RNA knockdown activity, Cas13b ortholog from *Prevotella sp*. (PspCas13b) was found to be more efficient and specific in RNA binding and knockout applications. The adenosine deaminase acting on RNA (ADAR) family of enzymes mediates endogenous editing of transcripts via hydrolytic deamination of adenosine to inosine (Nishikura, 2010) (Figure 3c). These enzymes are capable of precise base editing in RNA. This system used to edit RNA transcripts was referred to as RNA Editing for Programmable A to I Replacement (REPAIR). REPAIRv2 was further produced with higher specificity than other RNA editing platforms used previously (Stafforst and Schneider, 2012). REPAIR system is effectively used to mimic protective alleles that protect against several autoimmune diseases (Ferreira *et al.*, 2010). REPAIR presents a promising RNA editing platform with broad applicability for research, therapeutics and biotechnology.

### Application of base editors in crop improvement

Several agriculturally important traits are conferred by SNPs in the genome, and base editing has played a critical role in correcting those point mutations and accelerating crop improvement. Cytosine and adenine base editors have been successfully used in a wide range of major crops and model plants to edit specific genes conferred by single nucleotide polymorphisms (Hua *et al.*, 2018; Li *et al.*, 2018; Lu and Zhu, 2017; Ren *et al.*, 2018) (Table 2).

**Table 2:** List of genes targeted by cytidine and adenine base editors in different crops

Crop name	Targeted genes	Type of base editor used	Functions	References
*Oryza sativa*	*NRT1.1B and SLR1*	CBE	Enhance nitrogen use efficiency	Lu and Zhu (2017)
	*C287*	CBE	Herbicide resistant	Shimatani *et al*. (2017)
	*OsPDS, OsSBEIIb*	CBE	Nutritional improvement	Li *et al*. (2017)
	*OsCDC48*	CBE	Regulate senescence and death	Zong *et al.* (2017)
	*OsSPL14*	CBE	Herbicide resistance	Tian *et al*. (2018)
	*OsMPK6*	ABE	Pathogen‐responsive gene	Yan *et al*. (2018)
	*OsACC‐T1*	ABE	Herbicide resistance	Li *et al*. (2018)
	*SLR1*	ABE	Della protein for plant height	Hua *et al*. (2018)
	*OsSPL14*	ABE	Plant architecture and grain yield	Hua *et al*. (2018)
	*OsRLCK185, OsCERK1*	CBE	Defence response	Ren *et al*. (2018)
	*Pi‐d2*	CBE	Blast resistance	Ren *et al*. (2018)
	*Wx*	ABE	Rice amylose synthesis	Hao *et al.* (2019)
	*GL2/OsGRF4, OsGRF3*	ABE	Grain size and yield	Hao *et al.* (2019)
	*ALS*	CBE	Herbicide resistance	Veillet *et al*. (2019)
*Triticum aestivum*	*TaLOX2*	CBE	Lipid metabolism	Zong *et al*. (2017)
	*TaDEP1, TaGW2*	ABE	Panicle length and grain weight	Li *et al*. (2018)
Zea mays	*ZmCENH3*	CBE	Chromosomal segregation	Zong *et al*. (2017)
*Solanum tuberosum*	*StALS, StGBSS*	CBE	Herbicide resistance, Starch synthesis	Zong *et al.* (2018)
*Solanum lycopersicum* and *S. tuberosum*	*SLALS1*	CBE	Herbicide resistance	Veillet *et al*. (2019)
*Citrullus lanatus*	*ALS*	CBE	Herbicide resistance	Tian *et al*. (2018)

ABE, adenine base editor; CBE, cytidine base editor.

### CBEs in crop improvement

Several studies have demonstrated the successful applications of cytidine base editors in wide range of plants including rice, maize, tomato, wheat, cotton and watermelon (Lu and Zhu, 2017; Qin *et al.*, 2019; Tian *et al.*, 2018; Zong *et al.*, 2017). A “base editing” system was developed by using rat cytidine deaminase enzyme (APOBEC1) fused to the N terminus of Cas9 (D10A) using the unstructured 16‐residue peptide XTEN as a linker (Lu and Zhu, 2017). The APOBEC1‐XTEN‐Cas9 (D10A) fusion sequence was constructed into a binary vector, under the control of the maize ubiquitin promoter (UBI). This CRISPR/Cas9‐xyr5APOBEC1 base‐editing system was then used to induce point mutations in two rice genes *NRT1.1B* and *SLR1* with agricultural importance (Lu and Zhu, 2017). *NRT1.1B* gene encodes a nitrogen transporter and SLR1 gene encodes a DELLA protein. Earlier studies showed that nitrogen use efficiency in rice was enhanced with a C to T substitution (Thr327Met) in *NRT1.1B* (Hu *et al.*, 2015) and reduced plant height with an amino acid substitution in or near its TVHYNP motif (Asano *et al.*, 2009; Hu *et al.*, 2015). The base‐editing system was used to target one site each from these two genes and C to T substitution was achieved at a frequency of 1.4%–11.5% while 1.6%–3.9% of the edited plants accounted for C to G substitution. Besides base substitutions, indel mutations were also observed in sequencing results and it may be caused by the Cas9 (D10A) that nicks the nonedited strand. Although UGI increases the efficiency of base editing, it was not used in the above study.

Multiple herbicide resistance point mutations have been introduced into rice plants through multiplex base editing (Shimatani *et al.*, 2017). A target‐activation‐induced cytidine deaminase (Target‐AID) system along with a construct comprising of either dCas9 or nCas9 fused to *Petromyzon marinus* cytidine deaminase (*PmCDA1*)1 and sgRNAs was used to target the desired gene. A point mutation in *Acetolactate synthase* (*ALS*) gene results in herbicide resistance in plants (Yu and Powles, 2014). In rice, the C287T mutation of *ALS* homolog gene results in an A96V amino acid substitution in the encoded protein that confers resistance to the herbicide imazamox (IMZ). The researchers using the Target‐AID based base editing to introduce similar point mutation in the *ALS* gene. As expected, spontaneous resistance mutations were observed regardless of Target‐AID treatment at a frequency of 1.56%, but the resistant lines obtained from *nCas9^Os^PmCDA1^At^* transformants induced 3.41% IMZ tolerance. While no off‐targets were detected, seven out of the 14 edited lines showed the ALS‐A96V mutation.

Genetic variations were efficiently induced in rice crop by using a CRISPR/Cas9 toolkit comprised of rBE3 and rBE4 (rice base editors) (Ren *et al.*, 2017). In this study, the researchers fused a codon‐optimized rat APOBEC1 gene and UGI gene of *B. subtilis* bacteriophage PBS1 to Cas9n gene at both ends. The resulted base editor rBE3 (*APOBEC1‐XTEN‐Cas9n‐UGI‐NLS)* was expressed under the control of the CaMV35S promoter in the rice leaf sheath protoplasts together with *OsCERK1*‐targeting sgRNA transcribed from a rice U6 promoter. Subsequently, the researchers further optimized the rBE system with human AID (hAID) mutant version termed hAID*∆ for introducing point mutations in rice, thereby extending the base‐editing efficiency (Ren *et al.*, 2018). rBE5 (hAID*∆‐XTEN‐Cas9n‐UGI‐NLS) base editor was first tested in rice leaf sheath protoplasts, targeting two important genes, that is *OsRLCK185* and *OsCERK1*. Sequencing results revealed distinct mutations with a high frequency of C to T substitution suggesting that rBE5 base editor functions well on GC, AC, TC as well as CC sequence contexts in rice cells. Subsequently, rBE5 was used to target *Pi‐d2*, an agriculturally important rice gene that harbour a point mutation modulating defence response to blast fungus (Chen *et al*., 2006). G to A conversion was detected in eight heterozygous lines with 30.8% mutation efficiency.

Zhou and his group further expanded the toolkit by fusing a UGI gene to the 3′ terminal of rBE5 resulting in pUbi: rBE9 vectors. rBE9 vectors were used to target four different chromosomal sites (*OsAOS1*, *OsJAR1*, *OsJAR2* and *OsCOI2*) in rice transgenic calli and evaluated its editing efficiency in different sequence contexts (Ren *et al.*, 2018). Sequencing results revealed that rBE9 functioned more efficiently on GC context and more efficiently on multiple target C in the editing window of sgRNA than rBE3, resulting in more genetic variation at the target loci. Overall, the study indicated that the hAID*∆‐based rBE5 and rBE9 vectors favour GC and function on AC, TC and CC as well. Considering the high GC content of the rice genome, the pUbi: rBE5 and pUbi:rBE9 vector systems could be suitably used for generation of both gain‐of‐function and loss‐of‐function mutants of rice with respect to several agronomically important traits. Further, the usage of these tool kits could be expanded into other monocot and dicot plants for molecular breeding in crops.

More recently, a new plant base editor, A3A‐PBE, was developed by using human APOBEC3A, fused to Cas9 nickase to further enhance the base‐editing efficiency in plants (Zong *et al.*, 2018). The third‐generation base editors, BE3, are successfully used to create C to T substitutions in various organisms. However, the editing window was limited to 5 nucleotide (nt) sequence and the editing activity was low in GC contexts. Thus, the previous base editor nCas9‐PBE (Zong *et al.*, 2017) was improved to create A3A‐PBE, where the rat APOBEC1 was replaced with human APOBEC3A whose codons were optimized for cereals. The efficiency of A3A‐PBE was tested in wheat and rice genes and C‐to‐T conversion was observed with increased efficiency (13.1%) than nCas9‐PBE. The editing window spanned a larger editing space of 17 nt and had a low frequency of undesired on‐target indels. The potato genes, *StALS* and *StGBSS,* were also targeted by A3A‐PBE. C‐to‐T conversion was observed in potato protoplasts with 11‐fold higher efficiency than nCas9‐PBE. The efficiency of A3A‐PBE was tested in different contexts, and it was observed that unlike nCas9‐PBE, A3A‐PBE edited cytosines equally well irrespective of any context. The study also indicated that A3A‐PBE fused with different Cas9 variants could potentially target 90% of the cytidines and guanidines in the rice genome.

In watermelon (*Citrullus lanatus*), transgene‐free herbicide‐resistant varieties were generated by using CRISPR/Cas9‐mediated base‐editing system (Tian *et al.*, 2018). The *ALS* gene encodes the enzyme that catalyses the initial step of the biosynthetic pathway for branched‐chain amino acids. Single‐point mutations at several conserved positions of *ALS* genes are known to confer high level of herbicide resistance in different plant species (Yu and Powles, 2014). The sgRNA was cloned into pBSE901, in which BE3, driven by double 35S promoter was used to convert C to T at 3–9 positions of the target on the watermelon genome. Further, the binary vector was transformed into cotyledons of watermelon ZG94 through *Agrobacterium‐*mediated transfer*. S*equencing results revealed C to T mutations at T0 generation with an editing efficiency of 23%. To test for herbicide resistance, two transgene‐free homozygous P190S plants together with wild‐type (WT) controls were treated with tribenuron, an herbicide which is highly effective on broadleaved weed control. The results demonstrated that all the wild‐type plants were severely damaged by tribenuron at 14 days after treatment, while the homozygous P190S plants were slightly affected and resumed normal condition within 30 days after treatment. Thus, the base‐editing system has been effectively used to generate non‐GM herbicide‐resistant varieties which have the potential to address the weed problems in watermelon.

Most recently, a study was conducted to target the *ALS* gene in tomato and potato plants by a CBE using *Agrobacterium‐*mediated transformation to get sulfonylurea herbicide chlorsulfuron‐resistant plants (Veillet *et al.*, 2019). The mutation of the Proline‐186 in tomato and potato *ALS1* gene confers chlorsulfuron resistance (Yu *et al*., 2010). The researchers designed one sgRNA targeting the *SlALS1* gene, ensuring that nucleotides encoding the Pro186 codon (CCA) were located in the edition window of the CBE. The guide RNA was cloned into the CBE binary vector and *Agrobacterium*‐mediated transformation was performed in tomato and potato plants. After 2 weeks of kanamycin selection pressure, plant tissues were transferred to a selective medium containing medium containing 40 ng/mL chlorsulfuron. 12.9% and 10% edited but transgene‐free plants were obtained in the first generation in tomato and potato, respectively. Chlorsulfuron‐resistant tomato plants were obtained with an editing efficiency of up to 71%. Thus, the co‐base‐editing of the *ALS* gene with another gene of interest in tomato and potato plants can reduce the deleterious effects of the random integration of the T‐DNA into the host genome and the transient expression of the base editor could limit the off‐target activity.

Cotton (*Gossypium hirsutum*) is an important fibre and cash crop which majorly contributes towards agricultural economy. Being an allotetraploid species, many alleles are highly homologous with a few SNPs. Thus, a precise approach like base editing is highly needed to create point mutations which will help in functional analysis of these homozygous alleles. In a recent study, a precise base‐editing system (GhBE3) has been developed by fusing cytidine deaminase domain (APOBEC) with nCas9 and UGI to create point mutations in cotton (Qin *et al.*, 2019). A binary vector construct (pRGEB32‐GhU6.7) was prepared to target two cotton genes *GhCLA* (a homologous gene to *AtCLA1*) and *GhPEBP* responsible for chloroplast development and multiplex branch developmental processes, respectively (Chen *et al.*, 2018; Mandel *et al.*, 1996). Sequencing results revealed C → T substitution with a high editing efficiency ranging from 26.67% to 57.78%. This study will be of great help in genetic improvement and functional analysis of the cotton genome.

### ABEs in crop improvement

Like CBEs, ABEs have also been used successfully in different crops for base editing. The ABEs used in mammalian cells have been well adapted and optimized to develop an adenine base‐editing system in plants to create point mutations (Hua *et al.*, 2018; Li *et al.*, 2018). ABE7‐10 is a highly efficient ABE that is used to convert A·T to G·C in a programmable manner in mammalian cells (Gaudelli *et al.*, 2017). ABE‐P1 (ABE plant version 1), the modified version of ABE7‐10, was used for precise A.T to G.C conversion in rice plants (Hua *et al.*, 2018). The editing efficiency of ABE‐P1 was tested in rice by targeting IPA1 (*OsSPL14*), an important gene for plant architecture in rice for the base editing. Previous reports say that a point mutation in the OsmiR156 binding site of *OsSPL14* perturbs OsmiR156‐mediated cleavage of *OsSPL14* transcripts, resulting in rice plants with an ideal architecture and enhanced grain yield (Jiao *et al.*, 2010). In this study, a sgRNA was designed to target the OsmiR156 binding sequence in *OsSPL14*. Out of 23 transgenic lines, 6 showed expected T.C substitutions at the target region with an editing efficiency of 26%. Nine predicted off‐target sites did not have any base‐editing events. The base‐editing window of ABE‐P1 (4‐7) in rice was broader than ABE7‐10 in mammalian cells, which has a 4 nucleotides base‐editing window. The results indicate the specificity and efficiency of ABEs in rice.

Furthermore, the efficiency of this adenine base‐editing system, ABE‐P1, was tested in rice by targeting the *SLR1* gene in rice, which encodes a DELLA protein. Previous reports say, point mutations in the DELLA and TVHYNP domains of SLR1 could block its GA‐dependent degradation, thereby reducing the plant’s height (Asano *et al.*, 2009). The researchers designed a second sgRNA (sgRNA2) targeting the TVHYNP domain of SLR1. Out of 40 mutated lines, 5 had an expected T‐C substitution at position 6 in the protospacer. A third sgRNA (sgRNAs3) was designed to target the OsmiR156 binding sites of *OsSPL16* and *OsSPL18 rice genes* simultaneously. Interestingly, two lines (SG3‐11 and SG3‐12) were simultaneously edited at *OsSPL16* and *OsSPL18*, demonstrating multiplex editing in rice. To further expand the scope of adenine base editing, the researchers replaced the SpCas9 (D10A) nickase and its sgRNA scaffold with the SaCas9 (D10A) nickase and a sgRNA scaffold matching SaCas9 in the pRABEsp‐OsU6 vector. The resulting base editor, ABE‐P2 could recognize a different PAM sequence, NNGRRT. To test the efficacy of pRABEsa‐OsU6sa vector, a fourth sgRNA (sgRNA4) was designed that simultaneously targets the OsmiR156 binding sites of *OsSPL14* and *OsSPL17* genes. Out of 31 transgenic rice lines, 14 lines harboured T‐C substitutions in the target site in *OsSPL14* and 19 lines had T‐C substitutions in the target site in *OsSPL17*. The base‐editing efficiencies were observed to be 45.2% and 61.3% at the *OsSPL14* and *OsSPL17* target sites, respectively, which are higher than those of pRABEsp‐OsU6 with sgRNA1. In summary, several sgRNAs were designed to test the efficiency and specificity of the adenine base‐editing system in rice. These ABEs have the ability to efficiently convert A.T to G.C in rice in a programmable manner. Also, the lack of indels or any form of mutations in both target and potential off‐targets witness the specificity of these base editors in rice. Overall, the study has broadened the scope of genome editing in rice and advanced precision molecular breeding of crops.

Similarly, ABE7.10 (base editors used in human cells) was adapted and optimized to an adenine base‐editing system in plants to create point mutations at multiple endogenous loci in rice and wheat (Li *et al.*, 2018). To develop an ABE system in plants, seven ABE fusion proteins, named PABE‐1 to PABE‐7, were created which varied in the position of the adenosine deaminase and the number and locations of nuclear localization sequences. Among them, PABE‐7 base‐editing construct, together with the sgRNA, was found to be efficient in inducing A to G substitutions with high fidelity at multiple loci in rice and wheat. The plant ABE system was further used to develop herbicide resistance in rice (Li *et al.*, 2018). A point mutation (C2088R) at the acetyl‐coenzyme A carboxylase (*ACC*) gene in *Lolium rigidum* provide broad‐spectrum resistance to herbicides (Yu *et al.*, 2007). Therefore, the plant ABE system was used to target the *OsACC‐T1* gene at C2186R position that corresponds to C2088R from *L. rigidum*. Out of 160 transformed lines, 33 harboured at least one T to C substitution in the target region with 20.6% mutation efficiency. The plant ABE system was also used to generate base‐edited plants in wheat by targeting TaDEP1 and TaGW2 genes. PABE‐7 and pTaU6‐esgRNA constructs were delivered into immature wheat embryos by particle bombardment and plants were generated. For TaDEP1 site, 5 heterozygous TaDEP1 mutant plants were identified harbouring an A to G substitution with four mutants heterozygous for TaDEP1‐A (tadep1‐AaBBDD) and one mutant heterozygous for TaDEP1‐B (tadep1‐AABbDD). For TaGW2 target site, 2 heterozygous mutants were identified with an A to G substitution at position 5 for TaGW2‐B (tagw2‐AABbDD). This is the first report of achieving A to G base‐edited plants in wheat and herbicide‐resistant rice plants. The expanded deamination window (4–8 of the protospacer) and high‐fidelity substitutions at the targeted loci with low indels make this plant ABE system a reliable tool for achieving targeted base editing in crop plants.

A to G conversion in rice has been facilitated by a fluorescence‐tracking ABE developed by using *E. coli* TadA variants and Cas9 variants (Yan *et al.*, 2018). The wild‐type *E. coli TadA* gene and the engineered TadA*7.10 were fused to Cas9n and dCas9 with two 32‐amino acid XTEN2 linkers, resulting in rBE14 and rBE15, respectively. Similarly, A142N and P152R mutations were incorporated into TadA*7.10 to generate TadA*7.8 to create two more rice base editors rBE17 and rBE18. Later on, rBE14, rBE15, rBE17 and rBE18 vectors together with a *sgRNA* and an *mGFP5‐ER* cassette were introduced to target the pathogen‐responsive phosphorylation site in the endogenous *OsMPK6* gene into rice cells to investigate the efficiency of the ABEs. No mutants were identified for rBE15, rBE17 or rBE18 except for rBE14 with 16.67% efficiency. Sequencing results showed a pure A to G conversion at protospacer position −15 indicating that all mutant lines were heterozygous or monoallelic with one *OsMPK6* allele carrying the desired Y227P substitution. The study indicates that rBE14, together with the other rBE vectors, has the potential to facilitate generation of DNA variations in rice for both functional genomics and crop improvement. The study also suggests that the TadA variant TadA*7.10 is more suitable for base editing of A to G in the rice genome. Overall, these findings suggest that a fluorescence‐tracking ABE along with the Cas9n‐guided TadA: TadA7.10 heterodimer, not only introduce an A to G conversion in rice efficiently but also makes it more convenient to select the base‐edited plants through detection of fluorescence.

The scope of base editing was expanded in rice by generating new adenine and CBEs with engineered SpCas9 and *S. aureus* Cas9 (SaCas9) variants (Hua *et al.*, 2018). A number of rice genes *like OsSPL14, OsSPL16, OsSPL17, OsSPL18, OsTOE1* and *OsIDS1* were targeted by newly created ABEs like ABE‐P2, ABE‐P3, ABE‐P4 and ABE‐P5. The CBEs (CBE‐P1 and CBE‐P3) were used to target *SNB* and *PMSS3* genes, respectively. It was also reported that adenine and CBEs can be simultaneously executed in rice. These new base editors with different Cas9 variants have increased the scope of base editing and could be useful in rice functional genomics research in rice and other crops in the future.

Most recently, a rice codon‐optimized ABE‐nCas9 tool was synthesized to induce targeted A∙T to G∙C point mutation in the rice genome (Li *et al.*, 2019). In this study, the rice codon‐optimized *ec*TadA XTEN‐TadA*7.10 was cloned into pHUN411 binary vector under the control of a maize ubiquitin promoter. The rice amylose synthesis gene *Wx* was targeted by this vector. *Wx‐mq* is a minor mutant allele that results in low amylose content in rice endosperm (Sato *et al*, 2002) and the *Wx‐mq* allele contains a point mutation (T to C) at position 595, resulting in the replacement of tyrosine by histidine at residue 191. A sgRNA (Wx‐sg) was designed, cloned into the pHUN411‐ABE vector and transformed into rice plants via *Agrobacteria*. 16.67% clones had the desired T to C conversion at position 5, and approximately 27.78% clones harboured the substitution at position 6. Further the editing efficiency was increased by generating the pHUN411‐ABE‐sg2.0 vector using an extended version of sgRNA. The Wx‐sg was fused into the ABE‐sg2.0 binary vector, and base changes were observed in 5 out of the 33 transgenic lines (15.15%). The editing efficiency of ABE vectors was also tested by targeting *GL2*/*OsGRF4* and *OsGRF3* genes, responsible for grain size and yield in rice. Out of 35 transgenic lines of the pHUN411‐ABE‐GL2‐sg, 4 harboured the targeted base mutations. The transgenic plants using both vectors were analysed for off‐target activity, and none of them showed any off‐target mutations. Thus, the study indicates that the plant ABE systems combined with the modified single‐guide RNA variants have the ability to expand the application of CRISPR‐Cas9 tools as well as advance precise molecular crop breeding.

The scope of base editing was expanded by development of novel ABEs using a Cas9 variant SpCas9‐NGv1 that successfully induced A to G base substitutions in endogenous sites of the rice genome (Negishi *et al.*, 2019). The SpCas9‐NGv1 includes a 7‐aa mutation in the PAM‐interacting domain and recognizes NG as PAM (Endo *et al.*, 2019; Nishimasu *et al.*, 2018). To study the function of ABE7.10‐nSpCas9 in rice, the researchers constructed a binary vector harbouring a single‐guide RNA (sgRNA) and the ABE7.10‐nSpCas9 expression cassette which was then integrated into the rice genome via *Agrobacterium*‐mediated transformation. Sequencing results revealed that ABE7.10‐nSpCas9 effectively induced A to G substitutions at NGG PAM target sites at the position 16 to 13 nt upstream from the PAM. Further, ABE7.10‐nSpCas9‐NGv1, a new ABE system harbouring the nickase type of SpCas9‐NGv1 instead of nSpCas9, was used to target the endogenous sites in rice. The sequencing analysis revealed that all the four sites with NGG, NGA, NGC and NGT, respectively, as PAM sequences showed A to G substitutions and these were also inherited to the next generation. The study indicates that these new ABE systems developed with Cas9 variants can be used as valuable tools for precise genome engineering in crops.

## Limitations of base editing

### Targeting limitations

Successful base editing requires the presence of a specific PAM sequence (NGG PAM for SpCas9) and the target base must be within a narrow base‐editing window (Gaudelli *et al.*, 2017; Komor *et al.*, 2016). This specific PAM requirement is a severe limitation which lowers the editing efficiency in plants. To broaden the PAM compatibility and expand the scope of base editing, several research groups have developed novel ABE and CBE base editors using Cas9 variants which recognize PAMs other than the NGG motif (Endo *et al.*, 2019; Hua *et al.*, 2018; Nishimasu *et al.*, 2018; Qin *et al.*, 2019; Wang *et al.*, 2019). These optimized base editors can improve the base‐editing efficiency and expand its scope in targeting different sites in crop plants.

### Size of catalytic window

Cytosine deaminase base editors can potentially edit any C that is present in the wide activity window of approximately 4–5 nucleotides (or up to 9 nt). This is a severe limitation in base editors which result in low specificity and editing efficiency. Therefore, efforts have been made to generate high‐precision base editors with narrow catalytic windows that can precisely edit a single cytidine residue within the catalytic window with high accuracy and efficiency (Tan *et al.*, 2019). These are developed by removing nonessential sequences from the deaminase and testing different proline‐rich linkers of specific lengths that can narrow down the catalytic window and improve accuracy. Thus, these highly precise base editors with high efficiency can be used as valuable tools for precision crop breeding.

### Off‐target editing

The CRISPR‐mediated base‐editing technology is a much more precise tool used for base conversions without any gene disruption. However, off‐target editing is still a major concern. In the base‐editing systems, off‐targets occur when additional cytosines proximal to the target base gets edited. The off‐target activity has been greatly reduced in human cells by generating a high‐fidelity base editor (HF‐BE3), by installing mutations into BE3 base editor (Rees *et al.*, 2017). However, in a recent study, it was observed that CBEs BE3 and high‐fidelity BE3 (HF1‐BE3) induce unexpected and unpredictable genomewide off‐target mutations in rice crop (Jin *et al*., 2019). These mutations were usually the C to T type of single nucleotide variants (SNVs). The study also indicates that to minimize the off‐target mutations, it is necessary to optimize the cytidine deaminase domain and/or UGI components. Furthermore, use of improved variants of CBEs, YEE‐BE3, could also be employed to minimize the off‐target edits in plants (Jin *et al*., 2019).

## Future perspectives of the emerging technology

In the last two years, several research groups have engineered SpCas9s, SpCas9‐NG variants and xCas9 variants to extend the Cas9 recognized sites and expand the scope of base editing in plants (Endo *et al.*, 2019; Hua *et al.*, 2018; Nishimasu *et al.*, 2018). In a recent study, the optimized BE4max, AncBE4max and ABEmax editors (Koblan *et al.*, 2018) were further upgraded by using codon‐optimized bipartite nuclear localization signals (bpNLS) and were used to target rice genes (Wang *et al.*, 2019). The base editors showed much higher editing efficiencies as compared to the previous known CBE and ABE editors. These optimized and improved base editors are valuable tools for molecular breeding of crops. Thus, new engineered variants need to be adopted to improve the existing CBE and ABE base editors and increase the editing efficiency and expand the scope of base editing in a wide range of crops in the future.

The plant ABE system has been well adapted and successfully used in a wide range of crops (Hua *et al.*, 2018; Li *et al.*, 2018; Yan *et al.*, 2018). However, there are ample opportunities for improving and extending the plant ABE system by using engineered Cas9 variants recognizing different PAM sequences (Kim *et al.*, 2017; Hu *et al*., 2018) or Cpf1 (Li *et al.*, 2018). Furthermore, the sgRNAs could be ligated with different aptamers (MS2, PP7, COM and boxB (Ma *et al.*, 2016; Zalatan *et al.*, 2015) to facilitate simultaneous base conversions (C‐T and A‐G) and correct point mutations related to important agricultural traits (Li *et al.*, 2018). Protein delivery of base editors results in the precise conversion of nucleotides with enhanced DNA specificity (Rees *et al.*, 2017). Thus, high‐fidelity plant base editors should be created and delivered through RNP delivery to establish DNA free strategy with enhanced specificity and reduced off‐target editing.

Directed evolution employs multiple rounds of mutation followed by selection to engineer biomolecules with novel functions and protein variants with improved abilities (Soskine and Tawfik, 2010). CRISPR‐X generates diverse libraries of localized point mutations in mammalian cells that can be applied to study and improve protein function (Hess *et al.*, 2016). Most recently, a CRISPR/Cas‐based‐directed evolution platform (CDE) was developed for plants to evolve the rice (*Oryza sativa*) SF3B1 spliceosomal protein for resistance to splicing inhibitors (Butt *et al.*, 2019). These mutant variants confer variable levels of resistance to splicing inhibitors. This directed evolution platform can be used to engineer crop traits for better performance and develop resistance to biotic and abiotic stresses. It offers possibilities for breeding climate resilient crops that can enhance global food security. Thus, base‐editing diversification strategies for direction evolution need to be explored in the future that can increase genetic diversity in plants.

The use of DNA base editors in correcting point mutations related to agricultural traits has already been demonstrated in several crops. However, RNA editing has not been used in plants yet. Currently, the REPAIR system enables A to I conversion in RNA editing. In the future, additional fusions of dCas13 with other catalytic RNA editing domains such as APOBEC could also enable C to U conversions (Cox *et al.*, 2017). Conversion of A to I may also be possible on DNA substrates by using catalytically inactive dCas9 or dCpf1, either through formation of DNA–RNA heteroduplex targets (Zheng *et al.*, 2017) or mutagenesis of ADAR domain (Cox *et al.*, 2017). The REPAIR system is used to correct disease‐relevant mutations in human but its use in plants is still not explored. Use of RNA editing may not be highly desirable in crop bioengineering as it requires stable expression of CRISPR base editors. However, it would be good for functional gene analysis. Thus, researchers may explore the applications of this system in crop plants in the near future.

## Conclusion

Base editing is a novel editing tool in the genome engineering toolboxes. It is an efficient genome‐editing approach which enables nucleotide substitutions in a programmable manner without the requirement of a DSB or donor template. In the last three years, cytidine and adenine deaminase‐based base editors have been successfully developed and used for the base editing in plants as well as in animals. Narrowing down the catalytic window and adopting the Cas9 variants to improve the existing CBE and ABE base editors can expand the scope of base editing in crop plants. These upgraded base editors and mutants of cytidine deaminase can increase DNA specificity and lower the off‐target activity. The highly precise base editors can be widely used in model plants and crops for precision breeding. The emerging base‐editing technology is still in its infancy and a lot of efforts are to be made to optimize and expand the scope of editing and increase its efficiency. Nonetheless, it is a novel editing approach which has the potential to modify crops precisely and accelerate crop improvement in the future.

## Conflict of interest

The authors declare that there is no conflict of interest.

## Authors’ contributions

RM and RKJ conceived and drafted the manuscript. KJ collaborated in the manuscript preparation and critically reviewed the manuscript. All authors revised and approved the final manuscript.
